# Community Case Study Article Type: Criteria for Submission and Peer Review

**DOI:** 10.3389/fpubh.2016.00056

**Published:** 2016-04-14

**Authors:** Matthew Lee Smith, Sue E. Levkoff, Marcia G. Ory

**Affiliations:** ^1^Department of Health Promotion and Behavior, College of Public Health, The University of Georgia, Athens, GA, USA; ^2^Department of Health Promotion and Community Health Sciences, Texas A&M Health Science Center School of Public Health, College Station, TX, USA; ^3^College of Social Work, University of South Carolina, Columbia, SC, USA

**Keywords:** case study, review criteria, article type, intervention, evaluation criteria

## The Importance of Case Studies in Public Health Education and Promotion

Health programs and practices are often conceived and delivered by community-based practitioners to address specific community health education and promotion needs (1). Although, initially untested, such programs can provide important lessons for researchers and practitioners, alike. Given the growing emphasis on community-based participatory research (CBPR) approaches (2), it is especially important for researchers to build upon findings from CBPR studies, which can contribute to the development of evidence-based programs and practices for widespread dissemination (3).

While a community case study can take many forms (4, 5), we are defining it as a description of, and reflection upon, a program or practice geared toward improving the health and functioning of a targeted population. We utilize the term “community” in contrast to “clinical” studies, but it is important to note that a community can be defined in terms of geographic boundaries as well as demographic characteristics, common settings, and/or affiliations.

Typically, a community case study documents a local experience about delivering services to meet an identified need. Community-based studies often rely on community engagement principles, which are not typically incorporated in the more traditional science-based approach to evidence-based program development (e.g., CBPR, action research, and community-engaged research). The community case study that documents early experiences can contribute to programmatic development as well as to the future development of evidence-based practice. This has been referred to as the “practice to science” approach to the development of evidence-based practices (6). The community case study can also represent activities at later development stages, for example, documenting the experience of implementing an evidence-based program or practice in a different context (e.g., different culture, different population, and different setting) from that in which it was first developed [“from science to practice” (6)]. The lessons learned from such community case studies are essential for adaptation, replication, and eventual widespread dissemination and sustainability of innovations across a wide range of settings and populations.

Although case studies are a recognized form of research (5), the criteria for evaluating the quality of such efforts necessarily differs from empirical research articles where there is less attention to the local experience and context in which the intervention occurs, and more emphasis is given to the use of standardized research designs, measures, and analyses.

## Key Components of a Community Case Study

Under this article type, *Frontiers in Public Health Education and Promotion* will accept a broad spectrum of manuscripts that describe interventions, including programs and services, which promote public health education, practice, research, and/or policy. Such public health interventions can be implemented at the behavioral, organizational, community, environmental, and/or policy level(s). Articles require a description of the nature of the problem being addressed and rationale for the proposed intervention, the context (setting and population) in which the intervention is being implemented, and sufficient detail to allow replication of key programmatic elements. Reflections about public health impact as well as what works and what does not work should be highlighted. Additionally, submissions will require a discussion section that shares practical implications, lessons learned for future applications, and acknowledgment of any conceptual or methodological constraints. Articles should not exceed 5,000 words and include a maximum of five tables/graphs. Evaluation criteria for this article type are outlined below:

We recommend that community case study article submissions address the following issues (if relevant).

□ What is the problem? Whom does it affect?
□ What are the gaps about what is known or done currently?
□ What is the setting? Who are the key stakeholders? Who is the target population or participants?
□ With whom did you work or collaborate? Are there any unique characteristics of the team who worked to implement the solution?
□ What is the solution described by this community case study?
□ Is this solution innovative/novel in terms of content, format, and/or delivery? If yes, why?
□ What are the essential elements of the solution? Could this community case study be replicated? Include sufficient detail that the reader would know if replication would be feasible in his/her own context.
□ What are the barriers and facilitators to the development, implementation, and/or dissemination of the intervention?
□ What are the major successes of the solution? What are the promising results to date? Include data and/or evaluation results, if available.
□ How does this improve public health education, practice, research, and/or policy? What are the broader implications of this work?
□ Recommendations for those who want to replicate this in other settings, populations, or over time.

## Criteria for Review (Template for Review Editors to Complete for Each Manuscript)

Indicate what the community case study describes (check all that apply)

__an education effort
__a health promotion program
__a health promotion service
__an environmental change taking place in the community
__a technological change taking place in the community
__a policy change taking place in the community
__a community partnership
__others. Please specify: _____________________
__none of the above (i.e., inappropriately categorized for submission as a community case study article).

Indicate the target audience for the case study (check all that apply)

__educators
__community professionals
__health-care professionals
__lay public
__policy makers
__other. Please specify: _____________________

## Mandatory Sections and Associated Criteria

A community case study article has the following mandatory sections: abstract, introduction, background and rationale, description of the case, methodological aspects (including targeted population and setting), discussion, and lessons learned/recommendations. Are all sections present?

### Abstract

- Is the abstract written in a clear and comprehensive way?
- Does the abstract reflect major conclusions articulated in the case study?

### Introduction

- Does the introduction present the problem in an appropriate context?
- Other comments on introduction.

### Background and Rationale

- Is the intent of the case study adequately described?
- Is a justification made for the innovation/novelty of proposed case in content, format, and/or delivery?
- Are the questions asked by the case study most essential to the success of the initiative?
- Other comments on background and rationale.

### Essential Elements of the Intervention

- Is the intervention adequately described (e.g., development, previous findings if any, components, and format/design)?
- Is the intervention described in sufficient detail to understand the essential elements?
- Are the implementation procedures adequately described (e.g., how is the intervention being implemented in a particular setting, population, and/or partnerships; are any adaptations needed from prior work)?

### Methods

- Are the target setting(s) and population(s) adequately described so that context for the case study is clearly understood?
- Is this a single community or multiple community study?
- Is there an overall conceptual model or framework for understanding the importance of the problem and selection of intervention elements?
- Is it clear whether the emphasis is on furthering knowledge about the process and/or outcome of the case study? If focus is on process, is there attention to key elements of implementation such as reach, reproducibility, scalability, or sustainability? If on outcomes, are the metrics of success (outcome indicators) clearly articulated?
- Is the generalizability of findings/lessons learned addressed?
- Other comments on methods.

### Results

- Are findings/lessons learned accurately reported from data presented?
- Is the level of detail of the results appropriate (too much, too little, or about right)?
- Is any essential information missing?
- Other comments on results.

### Discussion

- Are the reported findings/lessons learned summarized briefly and described within the context of what is currently known about the public health issue(s) or problem(s) being addressed?
- Does the article conclude with practical recommendations for others who might replicate this intervention/program (or similar interventions/programs)?
- Does the article conclude with applied recommendations for those in the field who might deliver this intervention/program (or similar interventions/programs) in their communities/settings?
- Does the case study contribute concrete recommendations for delivering and/or improving the intervention for future applications (directed toward educators, researchers, or practitioners, as appropriate)?
- Does the article address any conceptual or methodological limitations for future implementation, dissemination, and sustainability?
- Other comments on discussion.

### Conclusion

- Are the conclusions justified?
- Overall, does the article contribute to building evidence-based practice and/or policy?

### References

- Is prior work, if any, properly and fully cited?

### Article Length

- A case study article should not exceed 5,000 words. Should any part of the article be shortened? If yes, please specify which part should be shortened.
- A case study article should not include more than five tables/figures. If there are more tables/figures included, please specify if you believe tables can be combined, condensed, or eliminated.

### Language and Grammar

- Are the language and grammar correct?
- Should the paper be sent to an expert in English language and scientific writing?

### Other Comments

- Please add any further comments you have regarding this manuscript.

## Reviewer Ratings

- Significance of issue being addressed by the case study: *scored out of a maximum of 10 points*
- Description of essential elements of the case study: *scored out of a maximum of 10 points*
- Appropriateness of the context (population and setting) in addressing the public health issue/problem described in the case study: *scored out of a maximum of 10 points*
- Sufficient conceptual and methodological detail describing why and how the intervention was implemented: *scored out of a maximum of 10 points*
- Reflections on what worked and did not work in the design, implementation, and/or dissemination of the program: *scored out of a maximum of 10 points*
- Quality of the writing: *scored out of a maximum of 10 points*
- Quality of the figure(s) and table(s): *scored out of a maximum of 10 points*
- Significance of the findings/lessons learned: *scored out of a maximum of 10 points*
- Could this intervention be replicated by other educators, researchers, or practitioners?
  __Yes
  __No

## Author Contributions

All authors were integral in formulating and drafting the manuscript and associated criteria.

## Conflict of Interest Statement

The authors declare that the research was conducted in the absence of any commercial or financial relationships that could be construed as a potential conflict of interest.
